# Quantification of the Impact of Structure Quality on Predicted Binding Free Energy Accuracy

**DOI:** 10.1021/acs.jcim.5c00947

**Published:** 2025-06-30

**Authors:** Sudarshan Behera, David F. Hahn, Carter J. Wilson, Simone Marsili, Gary Tresadern, Vytautas Gapsys, Bert L. de Groot

**Affiliations:** † Computational Biomolecular Dynamics Group, 28282 Max Planck Institute for Multidisciplinary Sciences, 37077 Göttingen, Germany; ‡ In Silico Discovery, Janssen Research & Development, Janssen Pharmaceutica N. V., Turnhoutseweg 30, 2340 Beerse, Belgium; § In Silico Discovery, Janssen Research & Development, Janssen-Cilag, C. Río Jarama, 75, 45007 Toledo, Spain

## Abstract

Relative binding free energy (RBFE) calculations have emerged as a powerful tool in drug discovery, capable of achieving experimental-level accuracy. However, the accuracy is compromised by a multitude of factors, including the initial structure modeling. The current study contributes to the quantification of the impact of initial structure modeling on the accuracy across a diverse set of activity cliff pairs. Along with providing a quantitative relation between the resolution of the crystal structure and free energy accuracy, we also demonstrate the incorporation of a secondary solvation tool (SOLVATE) to increase the free energy accuracy, especially when crystal waters are missing. The study also evaluates the reliability of AI-predicted structures in RBFE calculations, showing their effectiveness in predicting RBFE directionality and assigning nominal resolutions to the predicted structures based on free energy accuracy. These findings provide a set of recommendations for the development of more robust RBFE protocols, informing the use of structural data, solvation techniques, and AI-predicted protein models in drug discovery.

## Graphical abstract

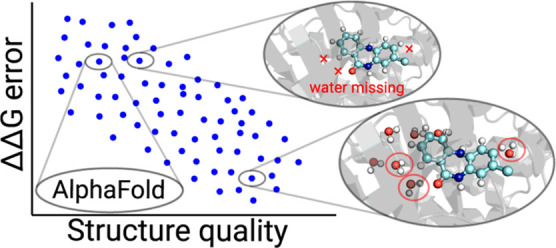

## 1 Introduction

Accurately quantifying the free energy difference between two states is invaluable in molecular biology and pharmaceutical research.^1–3^ This information provides crucial insights into fundamental biomolecular processes and serves as a cornerstone for rational approaches to protein engineering^4,5^ and drug design.^6^ Many important questions in biology can be addressed with free energy calculations with a proper definition of these two end states.^7^ A few examples are, protein–ligand absolute binding free energy (ABFE, the holo and apo states of the protein),^8–12^ relative binding free energy (RBFE, two chemically different ligands bound to protein),^3,13–15^ folding free energy change of a protein due to mutation or protonation (the wild type and mutant),^16,17^ etc. For this purpose, alchemical free energy methods^18–20^ prove exceptionally valuable. These techniques enable a smooth and chemically unrealistic transformation between the well-defined physical states and estimate the free energy difference between them.

The increase in high-performance computational facilities has enabled practitioners from both industry and academia to benchmark alchemical-free energy tools against large-scale data sets and apply them to real-world drug discovery campaigns.^11,13–15,21–23^ With the current state-of-the-art methodologies and workflows, RBFE uses a relatively small perturbation between the two states, meaning it tends to be a more robust, accurate and popular tool in drug discovery applications. It usually involves a difference of a few heavy atoms between two ligands, as compared to ABFE, where an entire ligand is created/annihilated. The gold standard for RBFE calculations is achieved when their accuracy matches experimental results within 1 kcal/mol.^24^ However, real-world applications often yield larger deviations, frequently exceeding 2 kcal/mol.^15,21,23^

Several key factors can compromise the accuracy of RBFE calculations: imperfect force field,^15,25,26^ insufficient sampling (and hence convergence),^27^ inadequate modeling of the starting structure,^28–38^ and/or errors in the experiments.^23,39^ Further, it is not easy to decouple the contributing factors, which can be highly interlinked. For example, an issue with force fields or initial structure modeling can lead to sampling problems. Although standard open-source small molecule force fields like GAFF2,^40^ CGenFF,^41^ and OpenFF^42^ as well as commercial force fields such as OPLS3/4 ^43,44^ can be used to obtain an average accuracy of 1 kcal/mol in benchmark data sets, many of the outliers in such benchmark studies have been attributed to an inadequate force field. While polarizable force fields (PFFs)^45,46^ and machine learning force fields (MLFFs)^47^ hold theoretical promise for improved accuracy over classical additive models, their empirical benefits in practical applications remain marginal to date.^45,48,49^ Recent works show that hybrid methods combining molecular mechanics with MLFFs like ANI-2x and mechanical embedding show no statistically significant improvement in accuracy.^48,49^ Moreover, these approaches involve significantly greater computational demands.

The limited prediction accuracy arising from insufficient sampling can be due to water displacement,^50^ large conformational changes^51^ upon ligand modifications, etc. The sampling problems can be tackled by incorporating enhanced sampling techniques or an increase in simulation length.^31,52,53^ The uncertainty involved in experimental values also contributes to large prediction errors associated with alchemical free energy estimates. It has been observed that the root-mean-squared error (RMSE) associated with experimental reproducibility is ∼1 kcal/mol,^23^ which sets the natural limit for the achievable accuracy for any affinity prediction method. Also, observations indicate that the discrepancy between predicted and experimental values is higher for larger experimental values.^15^

Another aspect which heavily impacts the accuracy of RBFE methods is the modeling of the initial structures. This includes the protonation states of amino acid residues^14^ and ligands,^36^ ligand binding pose,^33^ and treatment of water molecules.^30,31,34,35^ The protonation of amino acid residues is usually predicted using p*K*_a_ estimation tools like PropKa.^54^ Since RBFE calculations are performed mostly on congeneric series, the binding poses of ligands in the series are modeled using the crystal structure of one of the protein–ligand complexes, if available. Although the crystal waters are typically retained in the initial structure modeling in free energy simulations, some reports provide mixed results for retaining them during the system setup phase.^11^ Advancing free energy methodologies and protocols necessitates a comprehensive investigation to assess the impact of various factors on calculation accuracy. Such systematic exploration would facilitate the establishment of best practices and guidelines for these computations. Our current study contributes to this effort by examining the impact of initial structure modeling on the accuracy of free energy calculations across a diverse set of activity cliff pairs.

Activity cliffs represent pairs of structurally similar ligands that exhibit large differences in biological activity or potency against the same target.^55,56^ These cliffs represent a discontinuity in the structure–activity relationship (SAR), where small chemical modifications lead to substantial changes in activity. This phenomenon challenges traditional quantitative SAR (QSAR) models, often assuming a more gradual relationship between structure and activity. Activity cliffs can complicate drug discovery and optimization processes, as they may indicate potential pitfalls in predicting compound efficacy. Therefore, understanding and identifying activity cliffs can provide valuable insights into the optimization of lead compounds. Previously, Pérez-Benito et al.^31^ showed that the FEP+ protocol predicts activity cliffs with good accuracy (average unsigned error (AUE) from experiments 1.39 ± 0.07 kcal/mol) on a set of 33 freely accessible activity cliff pairs for 14 protein targets and 115 proprietary pairs across four different targets. They attributed the errors in some of the outliers to the difference in water placement and amino acid conformations in the ligand binding site by comparing the crystal structures of both the protein–ligand complexes of a pair.

The activity cliff data set^57^ is particularly well suited to a careful study of system setup because the crystal structures are available for the pair of ligands representing the activity cliff, and therefore, the end states can be prepared from and compared with the experimental structures. We systematically examine the impact of initial structure modeling on RBFE calculations performed using nonequilibrium alchemy. By leveraging a diverse set of crystal structures across varying resolutions, we establish a valuable mapping between resolution and RBFE accuracy. Our observation reveals that the solvation tool SOLVATE^58,59^ is surprisingly accurate in predicting crystallographic water molecules, achieving enhanced RBFE accuracy. Furthermore, the study benchmarks AI-predicted structures from AlphaFold2 (AF2) and AlphaFold3 (AF3) in RBFE calculations, showing their potential in the absence of a crystal structure. These results pave the way for more reliable and universally applicable protocols that will accelerate advancements in drug discovery and molecular design.

## 2 Methodology

### 2.1 Data Set

The activity cliff pairs for the current study were selected following a methodology similar to that of Pérez-Benito et al.^31^ The selected data set comprises 80 activity cliffs across 23 distinct protein targets, derived from the collection reported by Furtmann et al.^57^ In their analysis, Furtmann and colleagues employed a 3D similarity function^60^ that accounts for conformational, positional, and atomic property variations to classify ligand pairs as activity cliffs. Furtmann et al. applied stringent criteria, using cutoffs of 0.8 for 3D similarity and a 100-fold difference in potency to define activity cliff pairs. Additionally, they ensured that high-quality X-ray crystal structures (resolution <3 Å) were available for both protein–ligand complexes in each activity cliff pair. For our investigation, we selected only those pairs with a 3D similarity of at least 0.9. This data set provides an excellent opportunity to explore the structural origins of ΔΔ*G* errors in free energy calculations by enabling direct comparison between crystal structures of protein–ligand complexes. The names of the protein targets and their corresponding PDB IDs for the current data set are detailed in Table S1. Figure S1 shows the range of experimental RBFE values, which spans from 2.7 to 6.6 kcal/mol.

### 2.2 Simulation Setup

The protein–ligand complex structures, with a total of 113 PDB IDs, were extracted from the protein data bank. The missing residues and atoms were modeled using the pdbfixer tool,^61,62^ followed by the protonation of amino acid residues using PropKa^54^ at pH 7.0 and in the presence of the ligand. The ligand was removed from the complex for topology generation of the apo protein. The atoms and residues were renamed, wherever necessary, to match the naming convention of GROMACS.^59^ Then the N and C termini of the protein were capped with ACE and NME, respectively, with the help of pmx.^63^ The topology files for the proteins were generated using the *pdb*2gmx tool of GROMACS, employing the AMBER99SB*-ILDN force field.^64–66^

The extracted ligand structures from the crystal structures were subjected to the ACEPREP Web server^67^ to generate the protonation state, followed by manual inspections of the structures. The protonation states of a few ligands were adjusted in VMD^68^ by adding hydrogens to reflect chemical intuition; for example, a solvent-exposed aliphatic nitrogen atom was modeled as NH_3_^+^. The GAFF2 force field parameters^40^ were then generated using the ACPYPE^69^ and Antechamber^70^ tools with AM1BCC^71^ partial atomic charges. A virtual particle containing a small positive charge was attached to the chlorine and bromine atoms following the GAFF rules to represent the σ-hole.^72^

pmx^63^ was used to obtain the hybrid structure and topology for every pair of ligands. pmx creates the mapping between two ligands by finding the maximum common substructures, followed by distance-based atom mapping. Subsequently, hybrid structures and topologies were created. The direction of ligand transformation was chosen such that the more potent ligand (ligand-1) represents state A, leading to always a positive experimental ΔΔ*G*. The decision to enforce positive true ΔΔ*G* values was motivated by avoiding disjoint data clusters in quadrants II and IV of experimental vs simulation scatter plots. Without this adjustment, two distinct clusters would appear at magnitudes of >2.5 kcal/mol (experimental vs simulated ΔΔ*G*), with no data points near the origin (true ΔΔ*G* ∼ 0), creating a misleading visual impression of strong correlation between experiments and simulations. The rationale was visual clarity; the results should not depend on the directionality of ΔΔ*G*. The crystal structures containing ligand-1 and ligand-2 are called CS-1 and CS-2, respectively. For each pair of ligands, two hybrid structures were generated using the conformations of ligand-1 and ligand-2, separately. These hybrid structures were combined with their respective protein structures (from CS-1 or CS-2) for the free energy simulations. Finally, three sets of RBFE calculations, using either CS-1, CS-2 or both, were performed for each ligand pair (refer to Figure 1 for a schematic of the protocol).

**Figure 1:**
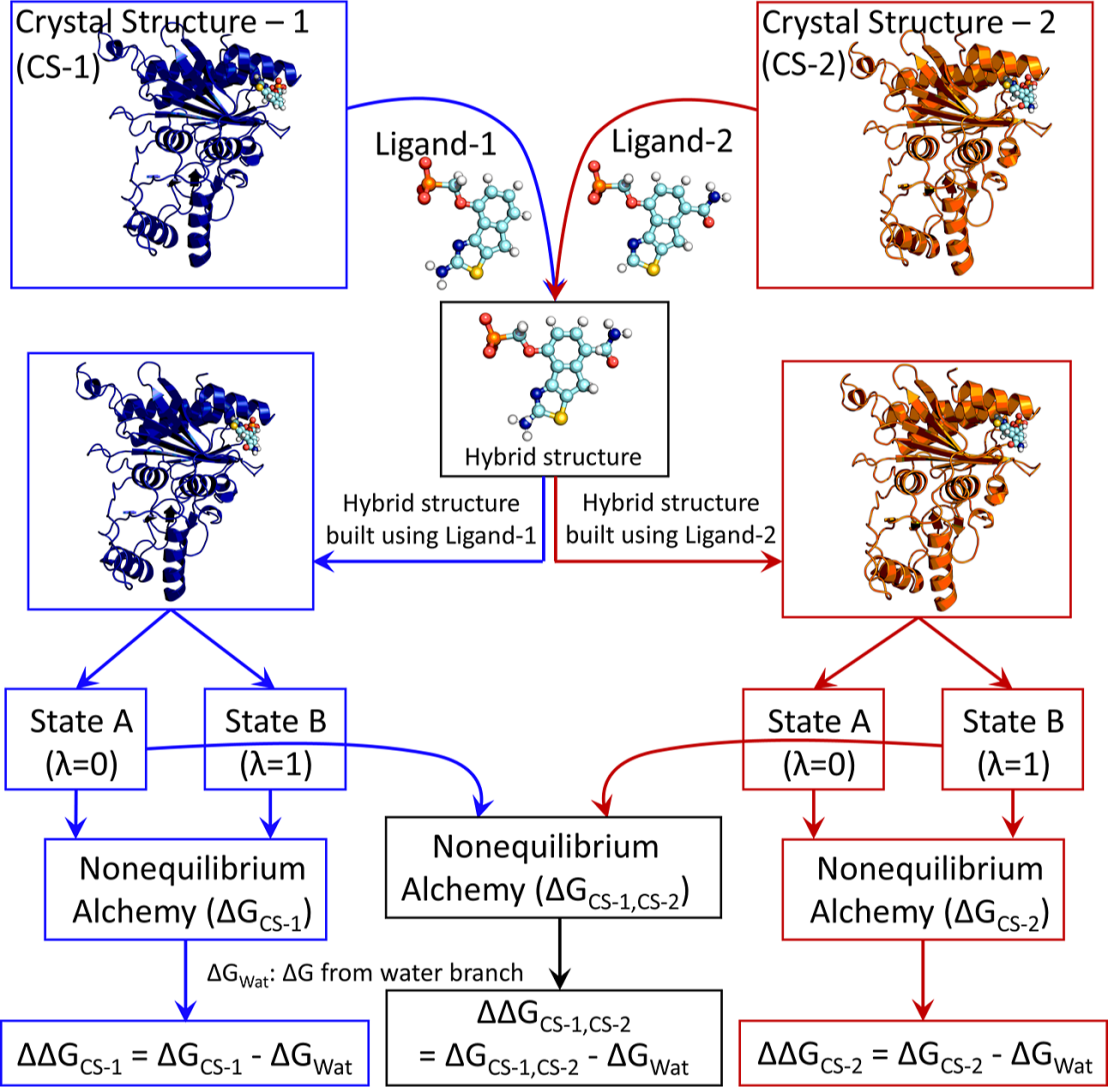
Schematic representation of the protocol employed in this study to calculate ΔΔ*G* using crystal structures CS-1, CS-2, or both. For details on the thermodynamic cycle used in the free energy calculations and a schematic on nonequilibrium alchemy, refer to Figures S2 and S3.

For the charge-conserving modifications, the ligand (for the water branch of Figure S2) and protein–ligand complex (for protein branch of Figure S2) were first placed in dodecahedron boxes, keeping at least 15 Å distance between the box wall and solute atoms. The charge-changing modifications were performed within the framework of double-system-single-box (DSSB) to maintain charge neutrality throughout the simulations.^7^ Both the ligand and protein–ligand complex were placed in a rectangular cuboid box by keeping the center-of-masses (COM) of these two systems along the longest axis. The two systems were positioned at least 30 Å and 15 Å from each other and from the box wall, respectively. The Cα atom closest to the COM of the protein and the ligand heavy atom closest to the COM of the ligand were position restrained with a harmonic potential of force constant 1000 kJ mol^–1^ nm^–2^, in the DSSB approach, to avoid interaction between them during the simulations. The boxes were then solvated with the TIP3P water model,^73^ followed by the addition of Na^+^ and Cl^–^ ions (with Joung & Cheatham’s ion parameters^74^) to neutralize the system and reach 150 mM salt concentration. For every ligand pair, both the physical states (state A and state B) of the ligands were simulated separately by setting the coupling parameter, λ, to 0 and 1. For the DSSB cases, the complexed ligand and solvated ligand present in the same box were simultaneously simulated in alternate states (state A for bound and state B for solvated ligand, or vice versa). The simulation protocol is similar to that reported by Gapsys et al.^14^ The simulation systems were then subjected to energy minimization, followed by a short *NVT* equilibration of 10 ps and finally 6 ns of simulation in an *NPT* ensemble. The first 2.250 ns were discarded as further equilibration and 80 equidistant frames were extracted from the last 3.750 ns. Alchemical transitions were spawned using these frames to the alternative end state, i.e., 0 to 1 or 1 to 0. The durations for the transitions are 200 and 500 ps for charge-conserving and charge-changing mutations, respectively (refer to Figure S3 for the protocol of free energy calculation). To ensure statistical reliability, we executed the full computational pipeline, from initial energy minimization to final alchemical transformation, three times independently for each system in our data set.

The energy minimization was carried out with steepest-descent algorithm^75^ with 100 kJ mol^–1^ nm^–1^ of maximum force tolerance. Stochastic dynamics thermostat and Parrinello–Rahman barostat^76^ with coupling time constants of 2 and 5 ps, respectively, were used for the molecular dynamics with leapfrog stochastic dynamics integrator^77^ and a 2 fs time step. All bonds were constrained using the LINCS algorithm^78^ and PME^79^ was employed to treat the electrostatic interactions with a direct space cutoff of 11 Å. The van der Waals interactions were switched from 10 to 11 Å, and dispersion correction was added to both energy and pressure. The nonbonded parameters were modified with the GROMACS implemented Beutler soft-core potential,^80^ with parameters σ = 0.25, and α = 0.3, during the alchemical switching. The d*H*/dλ during the transition was saved every time step. All the simulations were performed using GROMACS-2022.6.

The analyses of free energy calculations were performed employing pmx. The evolution of d*H*/dλ was integrated to obtain the work value for the alchemical transitions. The free energy difference was obtained from the work distributions of the forward (λ: 0–1) and reverse (λ: 1–0) nonequilibrium processes using the maximum likelihood estimator^81^ based on the Crooks Fluctuation Theorem^82^ (refer to Figure S3 for a schematic). The reported ΔΔ*G* is calculated as the average from three replicas. The standard errors reported on various quantities, such as AUE and percentages, are calculated using bootstrapping unless stated otherwise. Molecular visualization was performed using VMD^68^ and pymol.^83^ The number of data points is referred to as “*n*” throughout the manuscript.

The final uncertainty on ΔΔ*G* is calculated as follows, (i) for every leg (protein and water) and every replica, the 80 forward and 80 reverse work values were bootstrapped *n*_boot_ times (bootstrapped samples) to calculate Δ*G*_b_ (bootstrapped Δ*G* values). (ii) A Normal distribution was constructed using the previously calculated Δ*G* value (using the 80 forward and 80 reverse work values without bootstrapping) and standard deviation of the Δ*G*_b_ values, from which *n*_boot_ samples were extracted.

1 $$\Delta G_{bi}∼N\left(\Delta G,\sigma_{\Delta G_{b}}\right),,i=1,...,n_{boot}$$

The *n*_boot_ Δ*G*_b*i*_ values from all the replica for a leg (protein/water) are pooled together into a set (*S*_l_), for which the standard error (SE) on Δ*G* for that specific leg is estimated as

2 $$SE\left(l\right)=\sqrt{\frac{variance\left(S_{l}\right)}{n_{replica}}},,l=protein/water$$

3 $$S_{l}={\left\{{\left\{\Delta G_{b,l,\left(k\right)}^{\left(i\right)}\right\}}_{i=1}^{n_{boot}}\right\}}_{k=1}^{n_{replica}},,b:bootstrapped$$

The final uncertainty on ΔΔ*G* is calculated as

4 $$SE\left(\Delta \Delta G\right)=\sqrt{SE{\left(protein\right)}^{2}+SE{\left(water\right)}^{2}}$$

The *n*_replica_ and *n*_boot_ are 3 and 1000, respectively.

### 2.3 Crystal Water Prediction Tools

We employed three different tools - SOLVATE,^58,59^ WarPP^84^ and WaterDock^85^ - to predict and model water positions. For SOLVATE, 10 Gaussians were used to represent the solvent surface and a solvent shell of at least 5 Å was created. All the other parameters were set to default values. WarPP^84^ places water molecules only in the ligand binding site, and the site is defined as the space within 6.5 Å of ligand atoms. It generates water positions based on interaction geometry previously derived from crystal structures. The water-less protein and ligand structures were uploaded to the WarPP web server and the positions of water molecules were predicted in the binding sites. In the WaterDock^85^ protocol, water molecules are docked into the binding site using AutoDock Vina.^86^ The low-scoring positions are removed followed by clustering to keep only the centroids’ positions. The water molecules were docked into a cubic box with an edge of 15 Å (default value) centered on the COM of the ligand.

### 2.4 Alphafold Structures

For Alphafold2 (AF2),^87^ the protein structures (apo) were predicted using ColabFold,^88^ whereas for Alphafold3 (AF3),^89^ they were modeled using the AlphaFold Web server. The structure with the best ranking was considered for further modeling and simulations. The holo states were modeled using these apo structures by first aligning them with the crystal structures (CS) and then transferring the coordinates of the ligands from CS to the predicted structures. These structures were then subjected to the same structure preparation protocol (hydrogen addition, termini capping, etc.) as the CS.

## 3 Results

### 3.1 Overall Accuracy

For each ligand pair, ΔΔ*G* was estimated using two structures (CS-1 and CS-2, Figure 1). The AUE with respect to experiments considering the consensus and all the values of these two estimates are 1.75 ± 0.13 (*n* = 80) and 1.87 ± 0.1 kcal/mol (*n* = 160, Figure 2a), respectively. Considering the values from either CS-1 or CS-2 with the least deviation from the experiment (“Best set” in Figure S4), the AUE is 1.29 ± 0.16 kcal/mol (*n* = 80). This accuracy aligns well with previously reported values for data points with similarly large experimental RBFE values (AUE of 1.37 ± 0.19 kcal/mol for the data points with experimental |ΔΔ*G*| > 2 kcal/mol (*n* = 49), refer to Figure S5).^14^ Further, both the “Best set” and consensus estimates compare well with previously reported results using FEP+ on a common subset of data (Figure S6).^31^ In their study of the common subset (*n* = 30), Pérez-Benito et al. reported an AUE of 1.12 ± 0.18 kcal/mol and 1.56 ± 0.18 kcal/mol using FEP+ for the “Best set” and consensus set, which aligns well with the current investigation with corresponding values of 1.15 ± 0.17 and 1.64 ± 0.18 kcal/mol. This finding corroborates earlier studies demonstrating comparable accuracy between open-source tools like GROMACS and pmx, and commercial software such as FEP+.^14^ Previous investigations^11^ have reported improved accuracy for ABFE by combining both the holo and apo structure in a single free energy calculation. However, we did not observe a significant improvement using crystal structures of both complexes (refer to Figure 1 for protocol), which is discussed in Section S1. In line with previous reports,^15,90,91^ we also observe a lower accuracy for ligand transformation containing the sulfonamide group (Section S2). Further, both charge-changing and charge-conserving ligand mutations provide similar accuracy (Figure S11), which can be attributed to a higher simulation time spent in the former.

**Figure 2:**
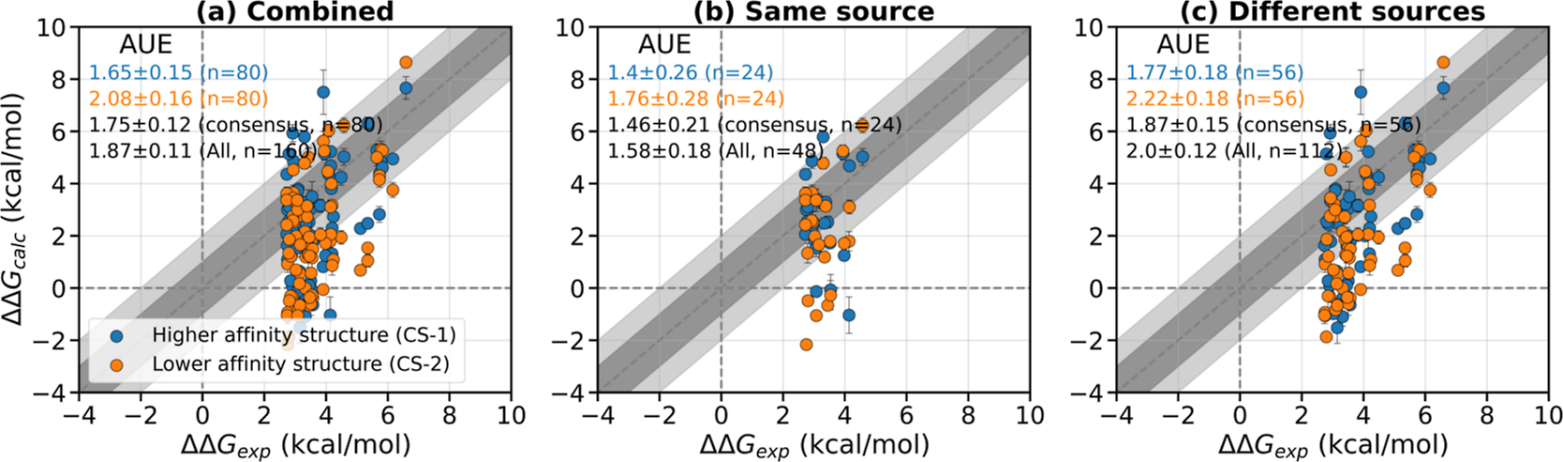
Comparison of calculated with experimental ΔΔ*G* values. (a) The two crystal structures are classified according to the relative affinity of the bound ligands as “Higher affinity structure (CS-1)” (blue) and “Lower affinity structure (CS-2)” (orange). AUEs (in kcal/mol) for these subsets, as well as the AUE for the ΔΔ*G*s of the consensus and considering all the data points, are written on the top-left corner. The data set is again split into two classes based on whether the experimental affinities are reported in the (b) same source or (c) different sources. The uncertainties were derived using bootstrapping.

Affinity emerges as a crucial feature differentiating the accuracy of predictions within structure pairs. We categorized each complex in a pair (CS-1 or CS-2) into either “Higher affinity” or “Lower affinity” subsets based on the relative binding strength of their ligands. The “Higher affinity” subset demonstrates significantly better accuracy (AUE: 1.65 ± 0.14 kcal/mol, *n* = 80, Figure 2a) compared to the “Lower affinity” subset (AUE: 2.08 ± 0.15 kcal/mol, *n* = 80). This observation can be attributed to the challenge of accurately modeling the environment of a higher-affinity ligand when starting from a lower-affinity state. For instance, forming a hydrogen bond present only in the higher-affinity ligand–protein complex starting from the lower-affinity structure is more challenging than breaking such a bond when starting from the higher-affinity structure.

The accuracy of the predictions is significantly influenced by the source of the reference experimental data. The data set was categorized based on whether the original affinity values for a given protein–ligand complex were reported in the same publication or derived from different sources. The results reveal a notable difference in accuracy depending on the consistency of the reference data source (Figure 2b,c). For “Higher affinity” complexes, the AUE is lower when the reference values originate from the same source (1.4 ± 0.26 kcal/mol, *n* = 24), compared to experimental data from different sources (1.77 ± 0.18 kcal/mol, *n* = 56). This trend is also observed in the “Lower affinity” and other combination subsets (Figure 2b,c). This variability in experimental data highlights the challenges in achieving high accuracy in computational predictions and emphasizes the need for careful consideration of data sources in model development and evaluation.

### 3.2 Structural Predictors of Accuracy

We investigated correlations between various structural features of the complex and the prediction error (UE). The explored features are resolution (Res), ligand average B-factor (Lig_Bf), number of water molecules within 5 Å of ligand (n_Wat), average B-factor of the protein residues present within 5 Å of ligand (Prot_Bf), and packing score of the ligand and protein residues present within 5 Å of ligand (Packing).^92^ Figure 3a,b illustrate the relationship between these features and the UE. All these features show mild correlations with UE. Figure 3a provides a rough quantitative framework for estimating the expected UE based on the resolution of a given complex. By considering the resolution of crystal structures, this relationship enables a practical assessment of the confidence in free energy calculations. B-factors and water occupancy are correlated to the resolution. Low-resolution crystal structures typically have fewer resolved water molecules and higher ligand and protein B-factors. Additionally, previous studies have shown that the packing score serves as a proxy for resolution.^92^ Further, a partial least-squares regression (PLSR) analysis (Figure S12) show only marginal improvement in correlation compared to using resolution alone.

**Figure 3:**
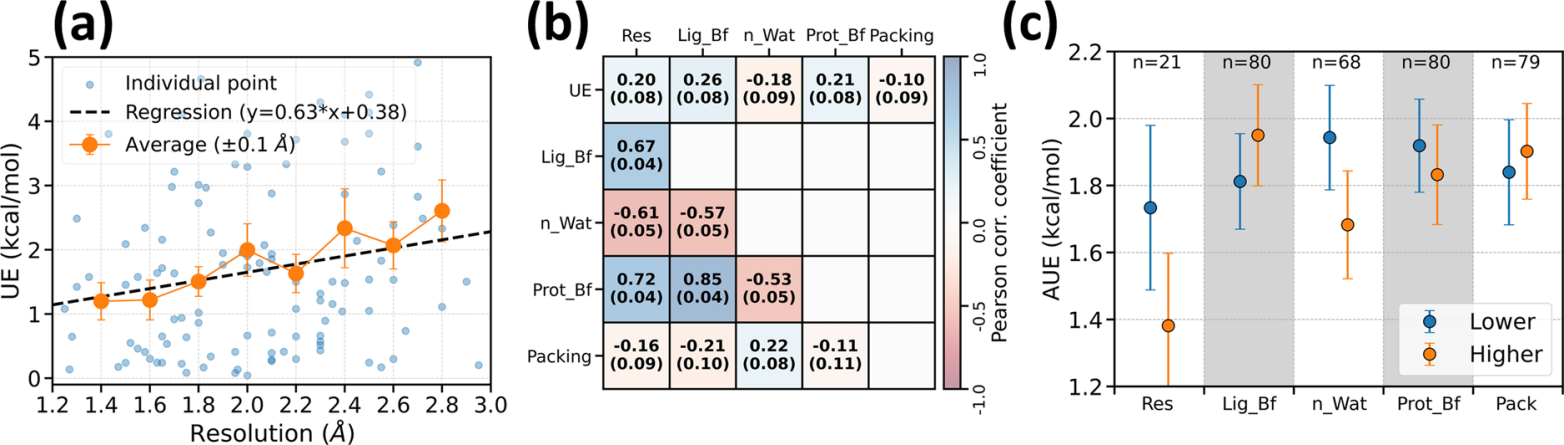
Analysis of prediction error in relation to structural features. (a) The dependence of unsigned error (UE) on the resolution of the crystal structure used for RBFE calculations. The data set comprises a total of 113 unique structures. The regression line was obtained by a linear least-squares fit. Each orange point represents the AUE of all the data points within ±0.1 Å resolution. (b) Pearson correlation coefficient matrix of different structural features with UE and with each other. Abbreviations used are “Res”: resolution, “Lig_Bf”: ligand average B-factor, “n_Wat”: number of water molecules within 5 Å of ligand, “Prot_Bf”: average B-factor for the protein residues present within 5 Å of ligand, and “Packing”: packing score^92^ for ligand and protein residues present within 5 Å of ligand. (c) The data set is split into two classes based on whether the crystal structure has a higher or lower value of the structural features, whenever different, and AUEs for these classes are plotted. For resolution (“Res”), a minimum difference of 0.4 Å was chosen to avoid noise. Figure S13 shows the change in AUE as a function of resolution difference. The uncertainties represent the bootstrapped standard errors.

The data set was further categorized based on the relative values of the structural features, whenever different, for each pair of complexes (lower and higher, as shown in Figure 3c). With a minimum difference of 0.4 Å, the higher resolution complexes have a lower AUE than the lower resolution structures. The change in AUE as a function of resolution difference is shown in Figure S13. Besides resolution, the number of water molecules in the binding site (n_Wat) has the largest impact on AUE. Other considered features contribute to negligible differences in AUE. Overall, the classification based on structural features highlights the crucial role of crystal water in the ligand-binding site of the initial structure. To address the issue of relevant and missing water molecules in the starting structure, we explored several water position prediction tools.

### 3.3 Role of Crystallographic Water

Water sampling is a critical factor in accurately estimating free energy differences using alchemical methods.^30,34,35,93^ The initial placement of water molecules can significantly impact water sampling, with incorrect initial positions potentially leading to inaccurate results. Crystal structures are often considered to provide reliable initial water positions. However, previous studies have reported mixed findings regarding the influence of crystal water positions on the accuracy of ABFE calculations.^11^ Here, we extend this investigation to RBFE calculations using the current data set. To assess the impact of crystal waters on RBFE accuracy, we repeated the entire free energy calculation protocol after removing all crystal water molecules from the crystal structures. The results reveal a significant impact of crystal water on the accuracy of RBFE calculations, particularly for the structures that gave the best performance compared to experiment, the “Best set”, where crystal waters are presumed to be better positioned than in other cases (e.g., compared to the “Worst set” in Figure S4). Removing the crystal waters from the “Best set” leads to a significant increase in the AUE from 1.29 ± 0.12 to 1.75 ± 0.13 kcal/mol (*n* = 80, Figure S14). A subset from the “Best set” was created, focusing on crystal structures where the presence of crystal waters (+CW) yielded a UE ≤ 1 kcal/mol relative to experimental values, and their removal (−CW) changed the UE by >1 kcal/mol compared to +CW. This subset represents cases where crystal waters significantly impact free energy estimates while their presence maintains high accuracy. The ability to predict the crystal water positions near the binding site (within 5 Å of ligand) by the GROMACS solvation tool *gmx solvate* was first tested on this subset.

Using the default scaling factor of 0.57 for van der Waals radii during water placement, *gmx solvate* accurately predicted only 29 ± 16% of crystal water positions in the binding site (Figure 4b). A predicted water molecule is considered correctly placed if it falls within 1 Å cutoff from a crystal water position in the ligand binding site. The low water position prediction accuracy explains the overall increase in AUE of ΔΔ*G* observed with −CW simulations of this subset (Figure 4d). While reducing the scaling factor improved prediction accuracy, it plateaued at 42 ± 13%. Interestingly, the default scaling factor of 0.57, which produces a density close to 1000 g/L for proteins in water, yields the best predictions after a short equilibration compared to other scaling factors. After a 5 ns equilibration, the accuracy of predicted water positions increases up to ∼60% (Figure 4b). This is still lower than the ∼80% retention rate of crystal waters after equilibration (horizontal solid lines in Figure 4c). It is important to note that this reduction of crystal water retention to ∼80% after an equilibration can be attributed to the rearrangement of their positions in the solvent environment.

**Figure 4:**
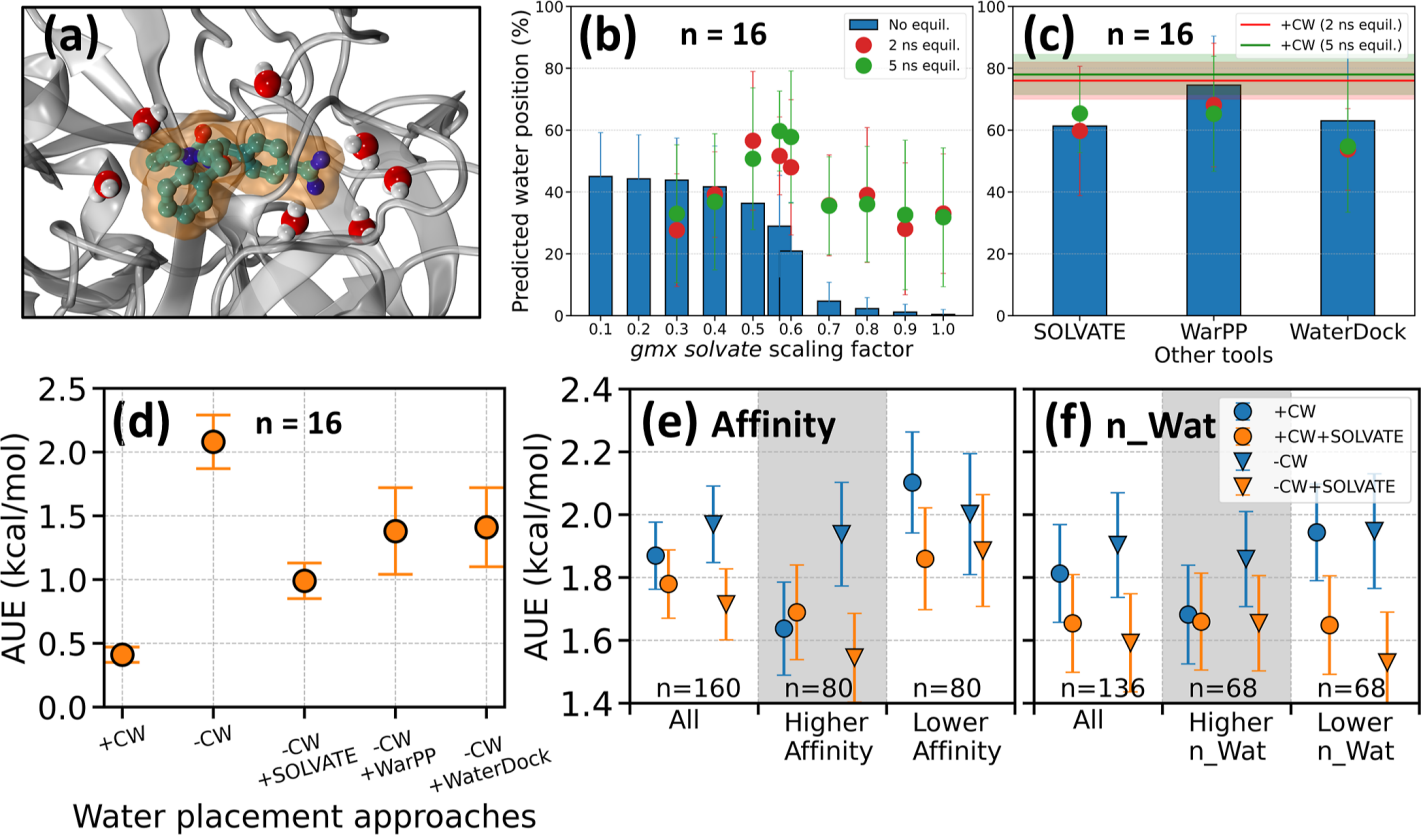
Impact of crystallographic water molecules on RBFE accuracy and evaluation of water prediction tools. (a) A snapshot showing water molecules present within 5 Å of ligand in the crystal structure of thrombin (PDB ID: 1ZHQ). (b) Percentage of crystal water positions predicted by *gmx solvate* using various scaling factors, including results after short equilibrations. A subset of the whole data set was used for this analysis, as explained in the main text. (c) Water position prediction percentages using SOLVATE, WarPP, and WaterDock for the same subset. Horizontal lines show prediction percentages after short equilibrations with crystal water present. Legend for histograms and spheres is consistent across panels b, and c. (d) AUE for the subset using different water prediction tools (SOLVATE, WarPP, WaterDock) after crystal water removal. (e,f) The entire set uses the SOLVATE tool, and the points are classified based on the affinity of the bound ligands and the number of water molecules (n_Wat) in the binding site. All cases in panels (d–f) use *gmx solvate* with the default scaling factor for the final solvation. The uncertainties are bootstrapped standard errors. The definition of “Higher” and “Lower” is the same as in Figure 2. Abbreviations used are “–CW”: without crystal water; “+CW”: with crystal water.

*gmx solvate* stacks a pre-equilibrated water box with the protein and removes clashing water molecules, and hence is not optimized for accurately predicting crystal water positions. To explore potential improvements, we investigated two well-benchmarked water placement tools (WarPP and WaterDock) and a hydration tool (SOLVATE). These tools demonstrated superior performance in predicting crystal water positions, with accuracies ranging from 60 to 75% (Figure 4c), outperforming *gmx solvate*. Notably, a brief equilibration period had minimal impact on these water position predictions. Considering the significant influence of crystal waters on the free energy accuracy of this subset, and the improved prediction capabilities of the additional hydration tools, their use might enhance free energy accuracy compared to *gmx solvate* alone, particularly in the absence of crystal water information. To test this hypothesis, we conducted free energy calculations on the same subset using these additional hydration tools in conjunction with *gmx solvate*. The results show that while these methods indeed outperform *gmx solvate* alone (“–CW” in Figure 4d), they still fall short of the accuracy achieved when using actual crystal water positions (+CW in Figure 4d). SOLVATE demonstrates superior performance compared to WarPP and WaterDock in terms of free energy accuracy.

The incorporation of SOLVATE was tested across the entire data set to further evaluate its effectiveness. While the overall improvement in accuracy was modest and statistically not significant, with the AUE for all structures decreasing from 1.87 ± 0.1 to 1.71 ± 0.11 kcal/mol (*n* = 160, “All” in Figure 4e), significant improvements were observed in specific subsets. SOLVATE demonstrated a more pronounced enhancement in free energy accuracy for compounds with lower relative affinity compared to those with higher affinity (Figure 4e). For structures where the crystal structure contains fewer water molecules in the binding site, the AUE decreased substantially from 1.94 ± 0.15 to 1.54 ± 0.16 kcal/mol (*n* = 68, “Lower n_Wat” in Figure 4f). This improvement was consistent regardless of the inclusion of crystal water in simulations, with slightly better accuracy observed without crystal water (“+CW + SOLVATE” and “–CW + SOLVATE”). Further, the accuracy for the subset with higher “n_Wat” is not affected by the incorporation of SOLVATE. These findings indicate that the relevant and missing water molecules in the “Lower n_Wat” set were effectively predicted by SOLVATE, achieving a level of accuracy comparable to the “Higher n_Wat”. The results also highlight the potential of SOLVATE to enhance free energy calculations by optimizing initial water placement, particularly in scenarios where crystal water molecules are missing, such as in AI-predicted protein structures (presented in the subsequent section).

### 3.4 Potential of AlphaFold-Predicted Structures in Free Energy Calculations

AlphaFold’s remarkable accuracy in protein structure prediction^87,89^ offers promising starting points for free energy calculations, particularly when experimental structures are unavailable. While some studies have successfully used AlphaFold-predicted structures and FEP calculations to reproduce experimental binding affinity data for various protein–ligand systems, including GPCRs, results have been mixed across different targets.^94,95^ To investigate this further, we evaluated the efficacy of using protein structures predicted by both AF2 and AF3 in RBFE calculations for all the protein targets in the current data set. This assessment aimed to gauge the viability of directly employing AI-predicted structures in free energy calculations without additional refinement. Since water molecules are missing from the predicted structures, SOLVATE was first used on these structures before the final solvation with *gmx solvate*.

The results, as shown in Figure 5a, reveal that the free energy accuracy achieved using AF2 and AF3 structures falls short of that obtained with crystal structures (CS). This discrepancy can be attributed to significant conformational differences between these predicted structures and CS, particularly in residue fragments surrounding the ligand (Figure 5b,c). For instance, certain fragments (Trp141-Gly155 and Glu229-Lys236) predicted as helices in AF2 and AF3 appear as loops in CS for thrombin (Figure 5b). In the case of Aurora-A (Figure 5c), both AF2 and AF3 predicted an open structure instead of the closed structure in CS. In some cases, like A4-h (Figure 5d), the predicted structures are in excellent agreement with CS and have lower AUE than CS. AlphaFold3 (AF3) generally outperformed AF2, with local conformations more closely resembling those in CS, especially for proteins like thrombin and HSP90-A (Figures 5b and S15a). The conformational differences between the predicted structures and CS could also stem from the fact that the predicted structures are in the apo state, with holo states modeled by aligning and transferring ligand coordinates from crystal structures. Future improvements may come from holo-structure prediction models like AF3, RoseTTAFold All-Atom (RFAA),^96^ NeuralPlexer1,^97^ UMOL^98^ and other cofolding methods.^99,100^

**Figure 5:**
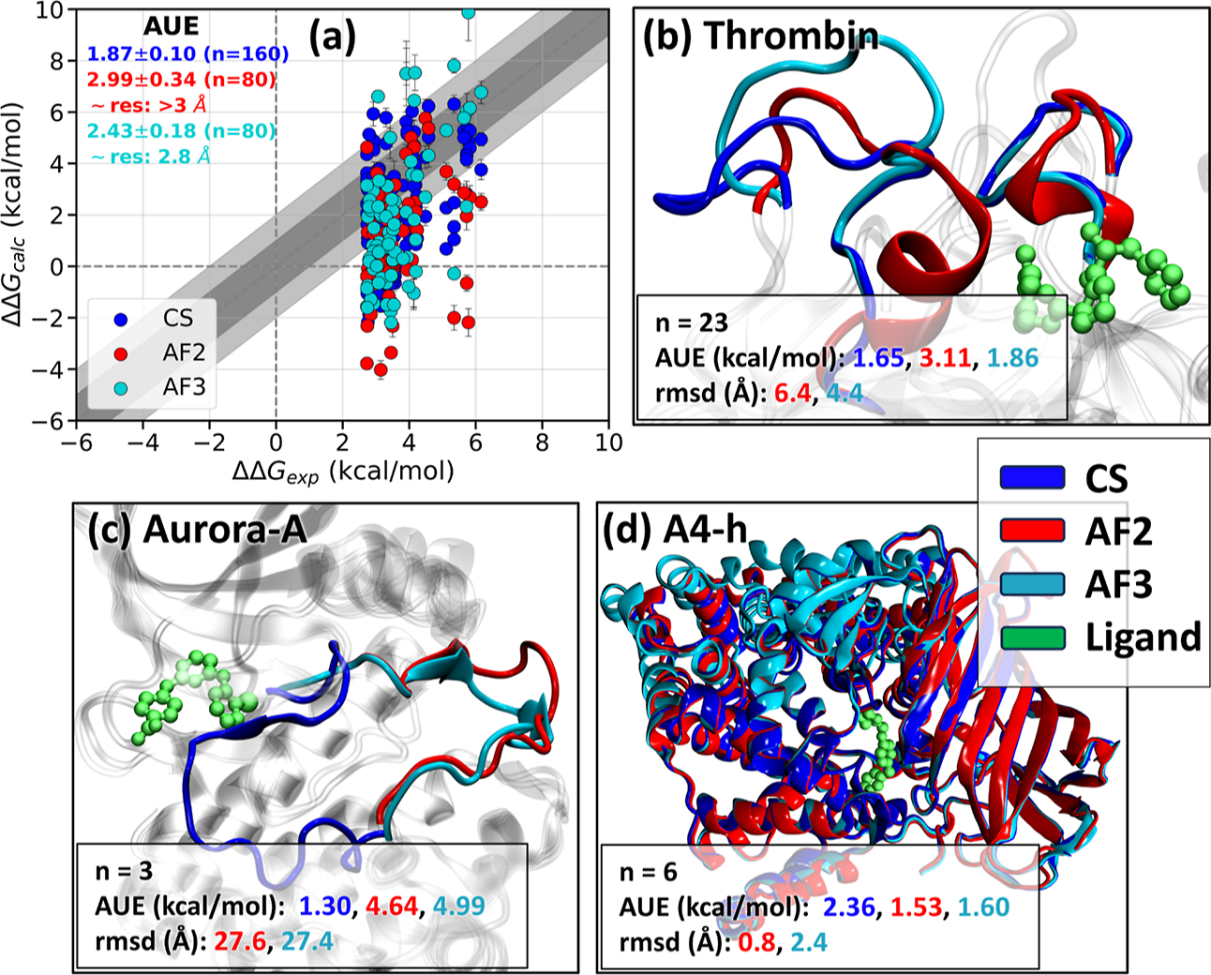
Comparison of using AlphaFold2 (AF2), AlphaFold3 (AF3) and crystal structure (CS) for RBFE accuracy. (a) ΔΔ*G* estimated using CS, AF2 and AF3 plotted against experimental values. The AUEs (kcal/mol) are written in the top-left corner. The nominal resolutions of AF2 and AF3 predicted structures based on the AUE and Figure 3a are >3 and 2.8 A, respectively. (b–d) Three example snapshots are shown highlighting the residue fragments near the ligand binding site for CS, AF2 and AF3. The number of data points (*n*), AUE and rmsd of the highlighted regions with respect to CS for AF2 and AF3 are listed. The uncertainty on AUE for panels (b–d), derived as bootstrapped standard error, varies from 0.25 to 0.5 kcal/mol. The color scheme is consistent across all panels. Abbreviations for protein target: Aurora-A—Serine/threonine protein kinase Aurora-A, A4-h—Leukotriene A4 hydrolase. Similar figures as panels (b–d) for two additional protein targets are shown in Figure S15.

We can use AUE between the calculated and experimentally measured ΔΔ*G* to assign nominal resolution to the predicted structures. Using the relationship between AUE and resolution (Figure 2a) we map the AUE of calculations using AF2 and AF3 predicted structures to a structural resolution of >3 Å and 2.8 Å, respectively. These nominal resolutions provide an estimate of the structural accuracy achievable by these predictions. It is important to note that these values are derived from the current data set, which includes 23 protein targets and 80 ligand transformations. These results may vary when applied to a larger data set. Further, both the AF2 and AF3 predicted structures exhibit the ability to predict the sign of the true ΔΔ*G* with an accuracy of 68 ± 5% and 73 ± 5% (82 ± 3% for CS, *n* = 80 for AF and *n* = 160 for CS), respectively.

## 4 Discussion

The current investigation on the effects of initial structural models on RBFE calculations revealed key findings with important consequences for future free energy calculations and computational drug design. The accuracy aligns well with previously reported values.^14,31^ It also demonstrates the effectiveness of nonequilibrium RBFE calculations^14,15^ in accurately predicting binding affinities for challenging activity cliff pairs, achieving an AUE of 1.75 ± 0.13 kcal/mol for the consensus set (*n* = 80, Figure 2a). The AUE is 1.38 ± 0.25 kcal/mol for the “Higher affinity” set if the reference affinity data is obtained from the same source (*n* = 24, Figure 2b). This level of accuracy is particularly noteworthy given the potential issues with the initial structures consisting of varying resolutions and the large differences in binding affinity that characterize activity cliffs, which often pose significant challenges for computational methods.

An interesting observation from our study is that structures containing higher-affinity ligands yield more accurate results in RBFE calculations (Figure 3c). This finding has important implications for the design of computational workflows in drug discovery. It suggests that initiating RBFE calculations from high-affinity complexes provides a more reliable starting point for exploring chemical space around a lead compound. However, it also raises questions about the potential limitations of this approach in scenarios where the goal is to predict large increases in affinity from a weakly binding starting point. The consistency of reference experimental data sources significantly impacts the accuracy. Our analysis reveals a notable difference in prediction accuracy depending on whether the affinity values for a given protein–ligand complex pair were reported in the same publication (AUE: 1.46 ± 0.22 kcal/mol for consensus set, *n* = 24) or derived from different sources (AUE: 1.87 ± 0.15 kcal/mol for consensus set, *n* = 56). The observed variability underscores the challenges in achieving high accuracy in computational predictions and highlights the importance of consistent experimental data sources. It suggests that when compiling data sets for RBFE calculations or benchmarking, affinity values from the same experimental source minimize potential inconsistencies and improve the reliability of their predictions. As noted previously,^39^ combining values from different sources can introduce significant noise, further emphasizing the need for data consistency.

The correlation between various structural features and RBFE calculation accuracy emphasizes the importance of high-quality structural data in achieving free energy accuracy (Figure 3). The relationship between resolution and UE provides a quantitative basis for assessing the potential reliability of RBFE predictions based on the resolution of available crystal structures (Figure 3a). This connection offers a practical tool to gauge the confidence level of the free energy calculations based on the resolution of available crystal structures lead optimization efforts.

Further, the number of water molecules present in the ligand-binding site is a significant structural determinant in distinguishing crystal structures that yield accurate free energy calculations from those that do not (Figure 3c). This highlights the importance of the accurate modeling of water molecules, which contribute to errors in free energy calculations^30,31,34,35^ and necessitates the development of improved water prediction and sampling methods. The removal of crystal water reduces the accuracy by ∼0.5 kcal/mol for a set of structures (the “Best set” in Figure S14). Our investigation into secondary water position prediction tools, including WarPP, WaterDock, and SOLVATE, revealed benefits in scenarios lacking crystallographic data (Figure 4d,e). The inclusion of SOLVATE in the protocol improved the free energy accuracy, especially in the cases where fewer water molecules are present in the ligand binding site (AUE improves from 1.94 ± 0.15 to 1.54 ± 0.16 kcal/mol, *n* = 68), suggesting its utility in compensating for incomplete or missing water information in starting structures. Given its demonstrated ability to improve free energy accuracy, particularly by effectively predicting and placing key water molecules in scenarios where crystallographic data is limited or absent (for example, in AI-predicted structures), the inclusion of SOLVATE in the free energy simulation protocol is strongly recommended to enhance the reliability of binding affinity predictions. Previous investigations revealed that presolvating the simulation box using Grand Canonical Monte Carlo (GCMC) water moves in FEP+ improves free energy calculation accuracy compared to methods without GCMC.^50^ Furthermore, this presolvation approach achieves accuracy equivalent to running GCMC moves during the free energy calculations themselves. From this observation, we speculate that this protocol could perform as well as or better than SOLVATE in predicting crystal water positions and free energy accuracy for the studied data set.

Leveraging AI-predicted (AF2 & AF3) protein structures, in conjunction with SOLVATE for water molecule placement, in RBFE calculations reveals both significant promise and important limitations. On average, both AF2 and AF3 structures yield lower accuracy than crystal structures (Figure 5a), which could be due to the apo conformation of the predicted structure. While our study used AF-predicted structures without explicit cofolding, recent analyses demonstrate that 67% of AF2 structures adopt holo-like conformations even in the absence of ligands.^101^ This suggests that AF predictions in our study may partially reflect holo-like features and may not resemble a true apo structure. AF3 consistently outperforms AF2, likely due to methodological advancements such as its expanded training data set and the integration of a diffusion network for improved structure prediction.^89^ Based on the relation between UE and the resolution of crystal structure (Figure 3a), the AF2 and AF3 structures refer to a resolution of >3 and ∼2.8 Å, respectively. Further, our results indicate that AI-predicted structures predict the directionality of ΔΔ*G* changes for RBFE calculations with accuracy closer to that with the crystal structures. However, it is important to note that AF is trained on these crystal structures, and hence the high accuracy obtained does not reflect the true accuracy achievable when applied to an unknown target in a prospective application. Further, the variability in performance observed across different protein targets (Figures 5b–d and S15) underscores the need for caution and further research. Identifying the key determinants of reliable RBFE results from AI-predicted structures is essential for enabling the confident and widespread adoption of these powerful tools in drug discovery and design.

## 5 Conclusions

In summary, our investigation highlights the critical influence of initial structural models on RBFE calculation accuracy. We show the significance of the resolution of the crystal structure, where a quantitative relationship with UE was established, providing an approximate estimate of prediction error based on resolution. Furthermore, the explicit inclusion and accurate modeling of crystallographic water molecules proved crucial, with SOLVATE demonstrating utility in water placement, especially when experimental data is limited. AI-predicted structures, such as those generated by AlphaFold3, provide practical alternatives when crystal structures are unavailable; however, their unpredictable performance with unknown targets in prospective applications and their inconsistent reliability across different targets require careful consideration. Based on our investigations, we recommend the following best practices to minimize prediction error: (i) use the protein structure bound to the most potent ligand available, (ii) incorporate the SOLVATE hydration tool into the workflow, particularly when water molecules are absent—as is the case with AI-predicted structures, (iii) select a lower resolution crystal structure if available, and (iv) ensure that experimental affinity data used for benchmarking comes from a consistent source. Overall, the current study provides key guidance for developing robust RBFE protocols to improve the accuracy of binding affinity predictions in future drug discovery campaigns.

## Supplementary Material



## Data Availability

The input files and ΔΔ*G* values can be found at https://github.com/deGrootLab/Initial_Structure_Modeling_RBFE_2025/. GROMACS and pmx are available freely at https://www.gromacs.org/ and https://github.com/deGrootLab/pmx, respectively.

## Notes

Additional figures and analysis highlighting the comparison with a previous study, use of both CS-1 and CS-2 in a single free energy calculation, the impact of sulfonamide group, and a table detailing the data set (PDF)
