# Supplementary material for: Quantification of the Impact of Structure Quality on Predicted Binding Free Energy Accuracy
Source: J Chem Inf Model. 2025 Jun 30;65(13):6927–38. doi: 10.1021/acs.jcim.5c00947 (PMC12264970; doi:10.1021/acs.jcim.5c00947)
Supplement: Supplementary file 1 [file ci5c00947_si_001.pdf]

Supplementary Information for: Quantification of the Impact of Structure Quality on Predicted Binding Free Energy Accuracy

Sudarshan Behera,† David F Hahn,‡ Carter J Wilson,† Simone Marsili,¶ Gary Tresadern,‡ Vytautas Gapsys,*,‡ and Bert L de Groot*,†

†*Computational Biomolecular Dynamics Group, Max Planck Institute for Multidisciplinary Sciences, Göttingen 37077, Germany*

‡*In Silico Discovery, Janssen Research & Development, Janssen Pharmaceutica N. V., Turnhoutseweg 30, 2340 Beerse, Belgium*

¶*In Silico Discovery, Janssen Research & Development, Janssen-Cilag, C. Río Jarama, 75, 45007 Toledo, Spain*

E-mail: vgapsys@its.jnj.com; bgroot@gwdg.de

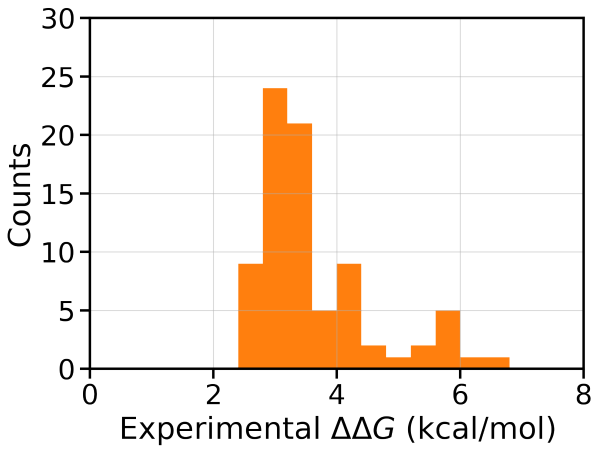

Figure S1: Distribution of experimental $\Delta\Delta G$ for the activity cliff pairs used in the current investigation.

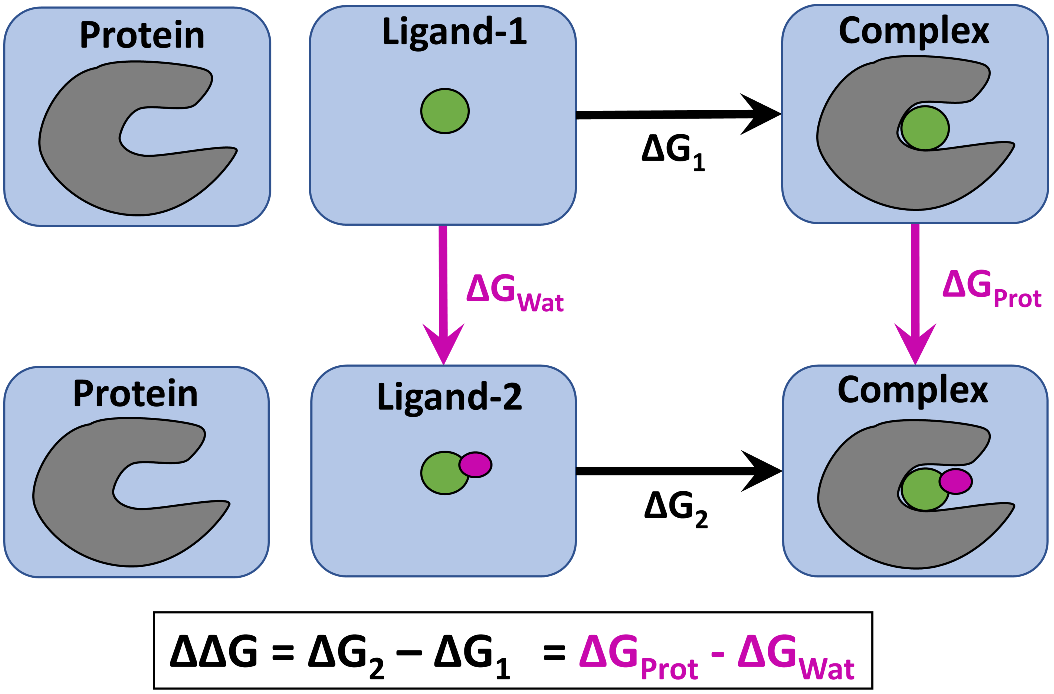

Figure S2: Thermodynamic cycle for the RBFE calculations. This schematic illustrates the alchemical transformation used to compute the difference in binding free energies between two ligands. Only the vertical legs are implemented using a nonequilibrium alchemical approach, which allows for an efficient calculation of $\Delta\Delta G$.

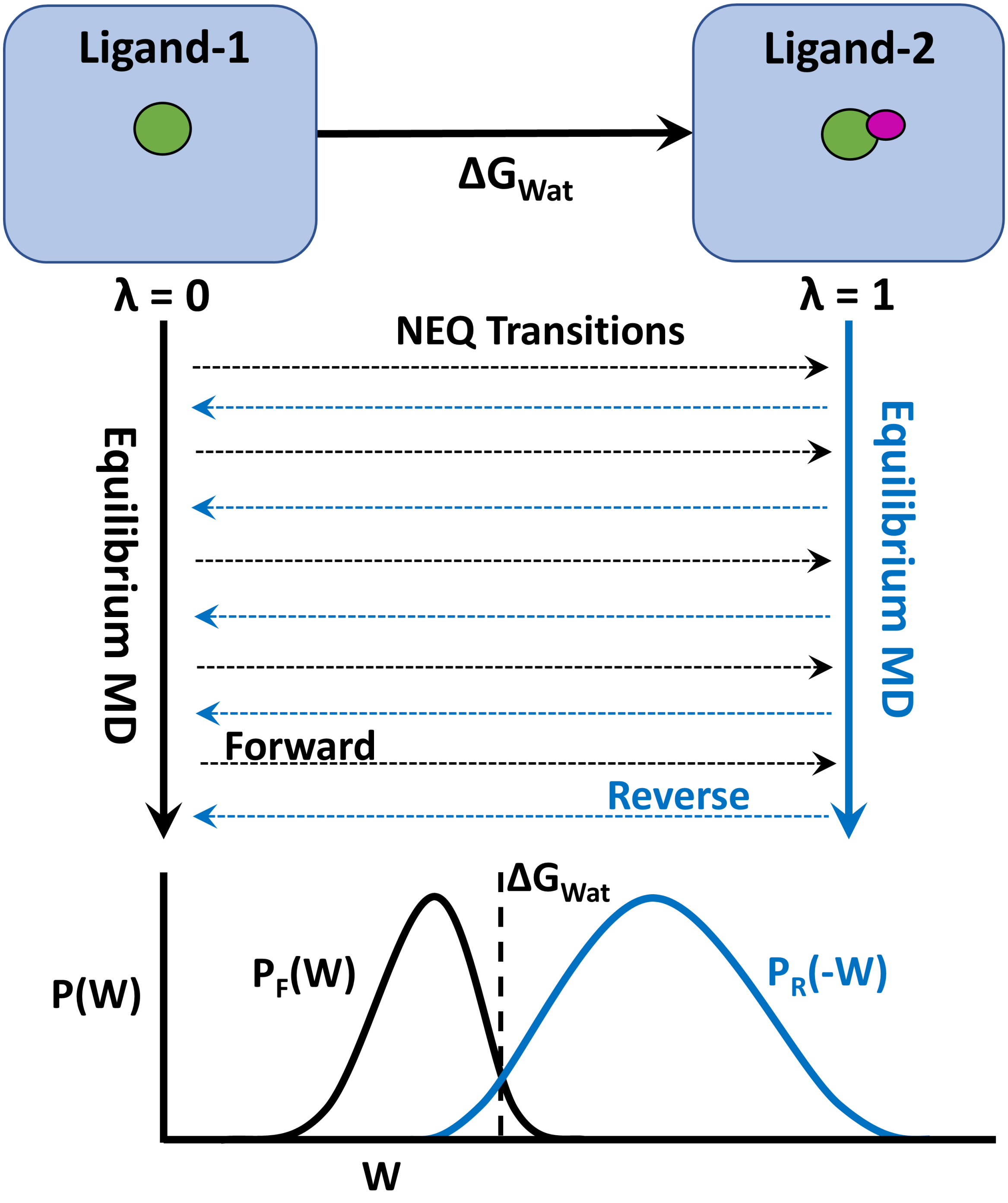

Figure S3: A standard protocol for the nonequilibrium alchemical approach is depicted for the estimation of ΔG_{Wat} (free energy change due to ligand mutation in water, Figure S2). Similar protocol is followed to obtain ΔG_{Prot}.

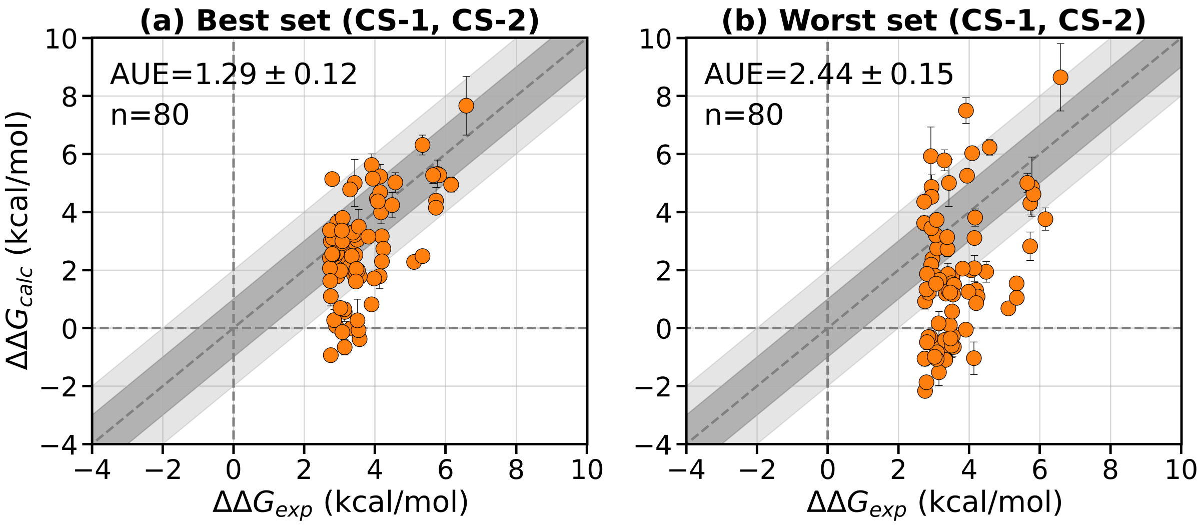

Figure S4: The dataset is divided into two subsets based on the UE from experiments. The 'Best set' and 'Worst set' represent the complexes out of CS-1 and CS-2 with the least and highest UE for each pair, respectively. The determination of whether a given CS is good or bad can only be made retrospectively; we have no way to assess this in advance or for prospective calculations.

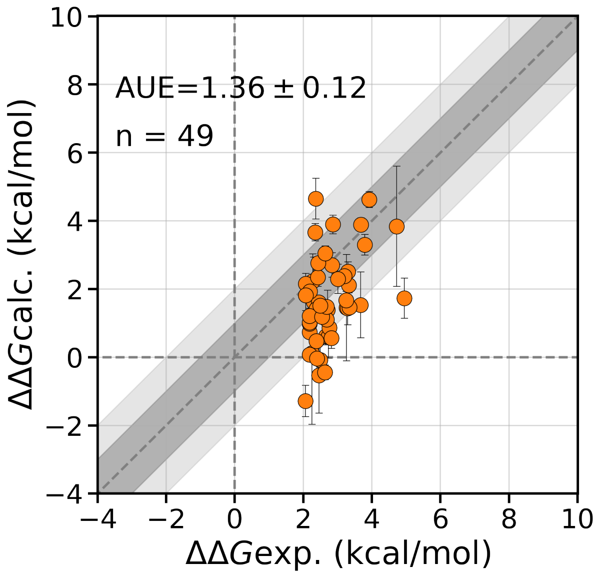

Figure S5: Comprison of the calculated with experimental $\Delta\Delta G$ values for ligand pairs with $|\Delta\Delta G_{Expt}| > 2$ kcal/mol. The data is obtained from Gapsys *et al.*[1] for GAFF2 forcefield.

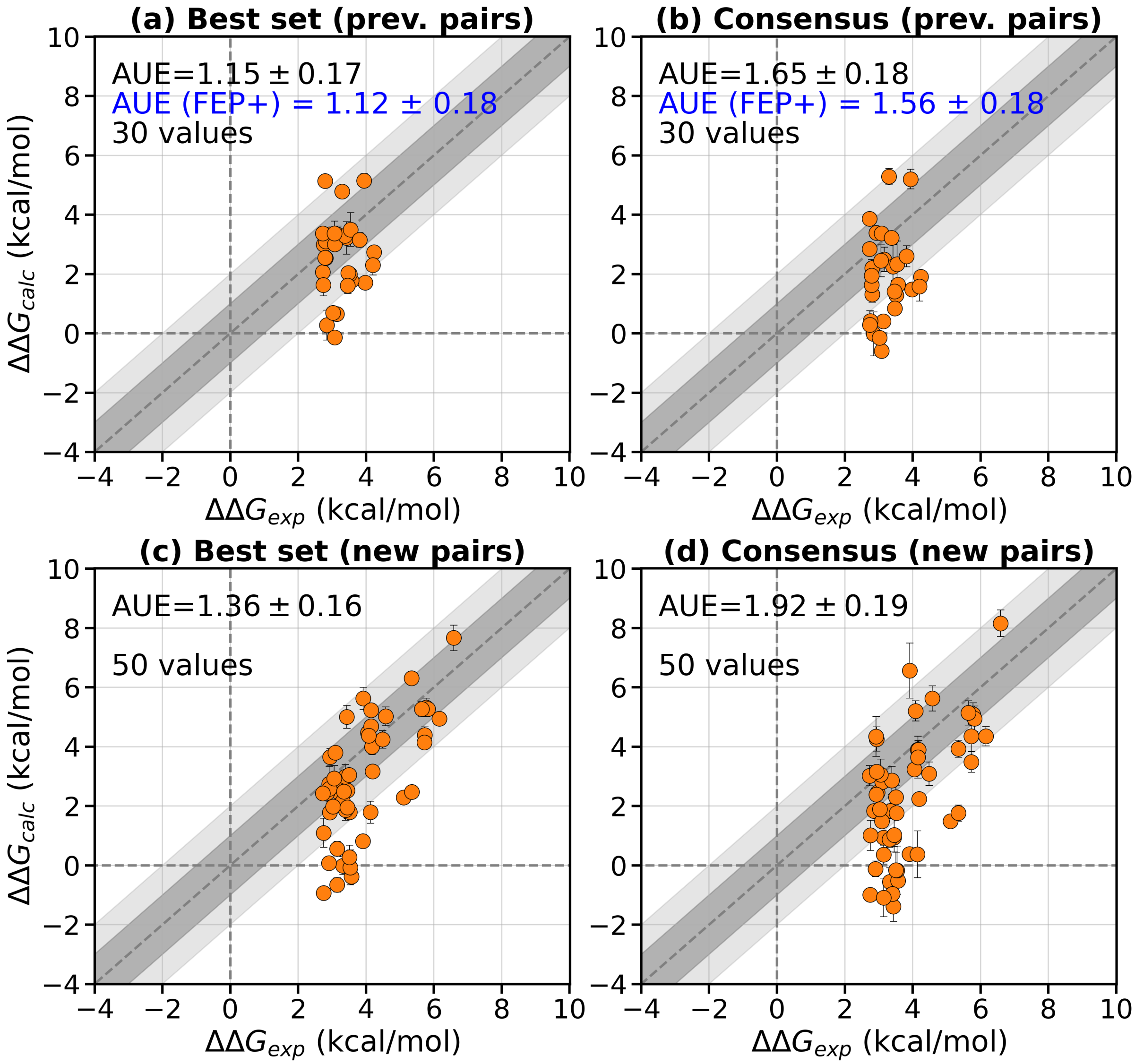

Figure S6: (a-b) Comparison of accuracy using a subset (prev. pairs) of the data, for which the $\Delta\Delta G$ values estimated using FEP+ are available from Pérez-Benito *et. al.*[2] The "Best set" follows the definition in the main text and the Figure S4, while the "Consensus" represents the arithmetic average of the estimates obtained using CS-1 and CS-2. The AUE obtained with the non-equilibrium alchemical approach employed in this work is comparable to that achieved with FEP+ protocol. (c-d) The same as panels a & b, but for the new activity cliff pairs.

S1 Combining both CS-1 and CS-2 in a single RBFE calculations

Non-equilibrium free energy calculation frameworks allow the use of distinct initial structures for the two end states, such as employing both holo and apo crystal structures for ABFE calculations. Previous investigations[3] have reported improved accuracy for ABFE with this approach. However, in our current investigation, we did not observe a significant improvement when using crystal structures of both complexes (CS-1 and CS-2) in a single free energy calculation (refer to Figure 1 for the protocol and Figure S7a for comparison with experiment). The AUE for this "Both" approach falls between those of using either CS-1 or CS-2. This outcome can be attributed to the fact that using both crystal structures only leads to substantial accuracy improvements compared to the "Best set" when there is a systematic deviation of the $\Delta\Delta G$ estimate from experimental values using either structure individually. The current dataset contains three ligand transformations where such an improvement is observed (Figure S8). Figure S9 illustrates the work distribution for one such case. Overall, we found that the $\Delta\Delta G$ estimate obtained using both CS-1 and CS-2 simultaneously is comparable to the consensus (average) of $\Delta\Delta G$ estimates calculated using CS-1 and CS-2 individually (Figure S7b).

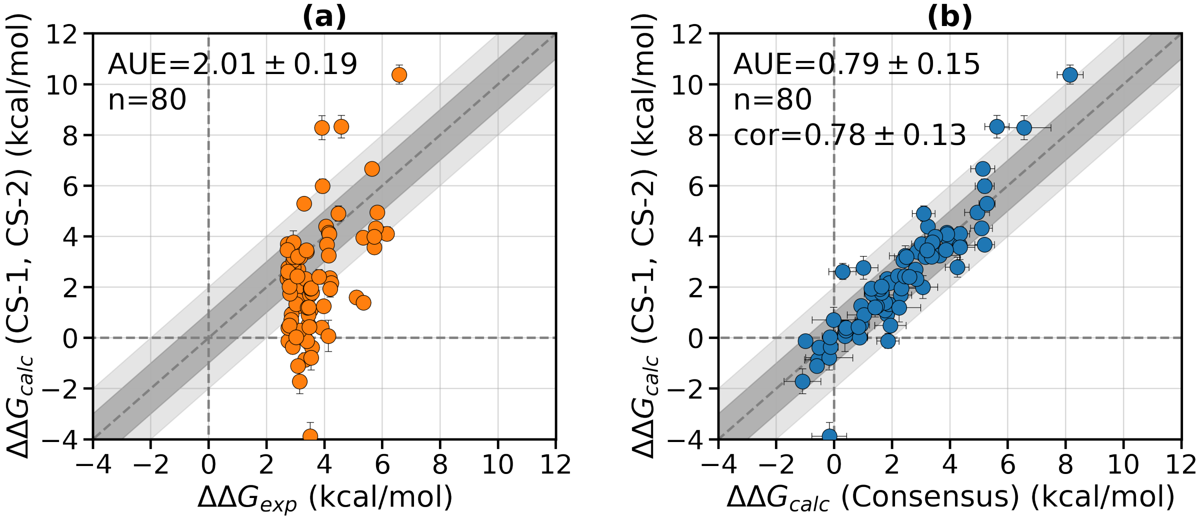

Figure S7: (a) Comparison between experimental $\Delta\Delta G$ values and the $\Delta\Delta G$ estimates obtained using both CS-1 and CS-2 in a single RBFE simulations. (b) Comparison of $\Delta\Delta G$ values calculated using this "Both" approach with the consensus $\Delta\Delta G$.

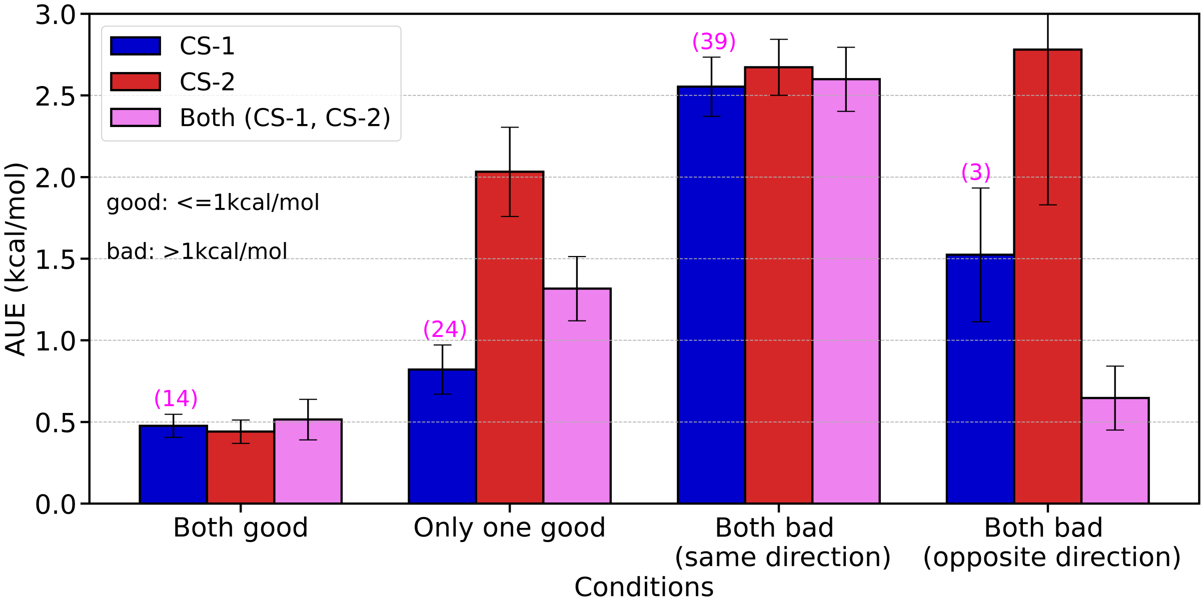

Figure S8: Comparative analysis of $\Delta\Delta G$ calculation accuracy using single versus combined crystal structures. Data points are categorized as 'good' (unsigned error (UE) ≤ 1 kcal/mol) or 'bad' (UE > 1 kcal/mol). The "Both" approach, utilizing CS-1 and CS-2 simultaneously, shows improved accuracy primarily when individual CS-1 and CS-2 calculations yield poor results with opposing signed errors relative to the experimental $\Delta\Delta G$.

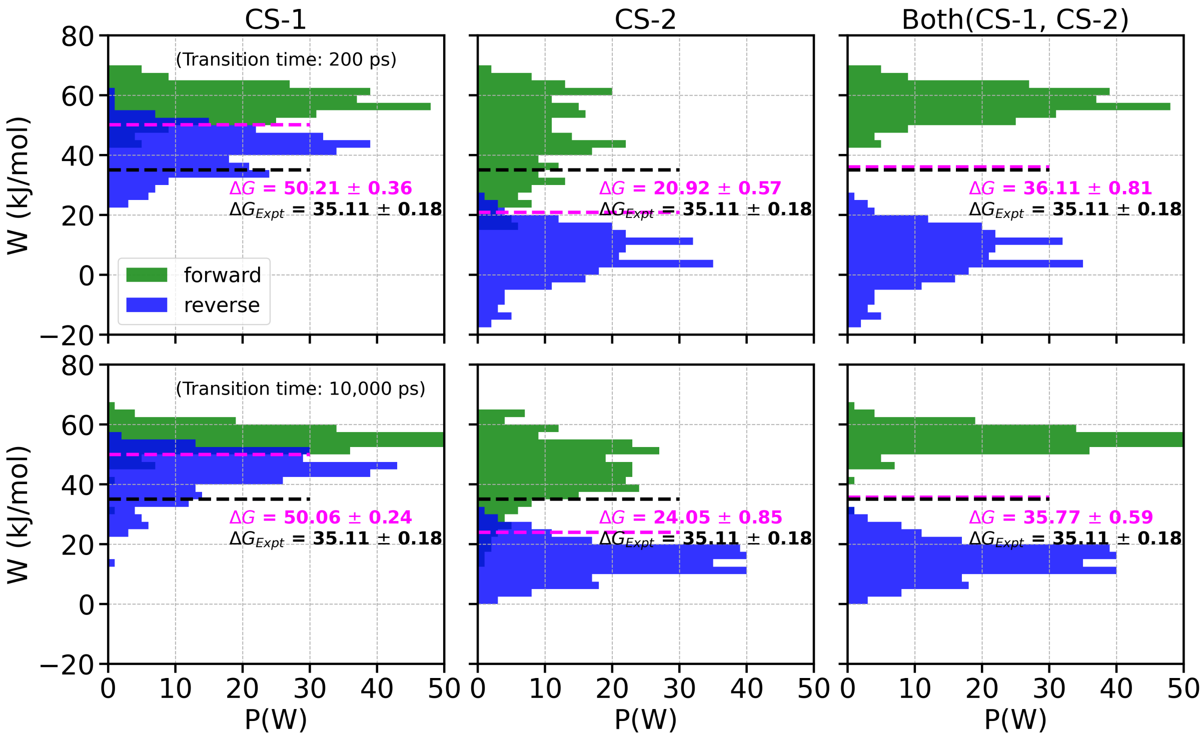

Figure S9: An example illustrating a scenario where ΔG_{Prot} estimated using CS-1 and CS-2 diverge in opposite directions from the experimental ΔG_{Prot} (ΔG_{Expt}). The ΔG_{Expt} is calculated as $\Delta\Delta G_{Expt}$ - ΔG_{Wat}. The top and bottom panels show the work distribution for 200ps and 10,000ps transition times, respectively.

S2 Impact of sulfonamide groups on RBFE accuracy

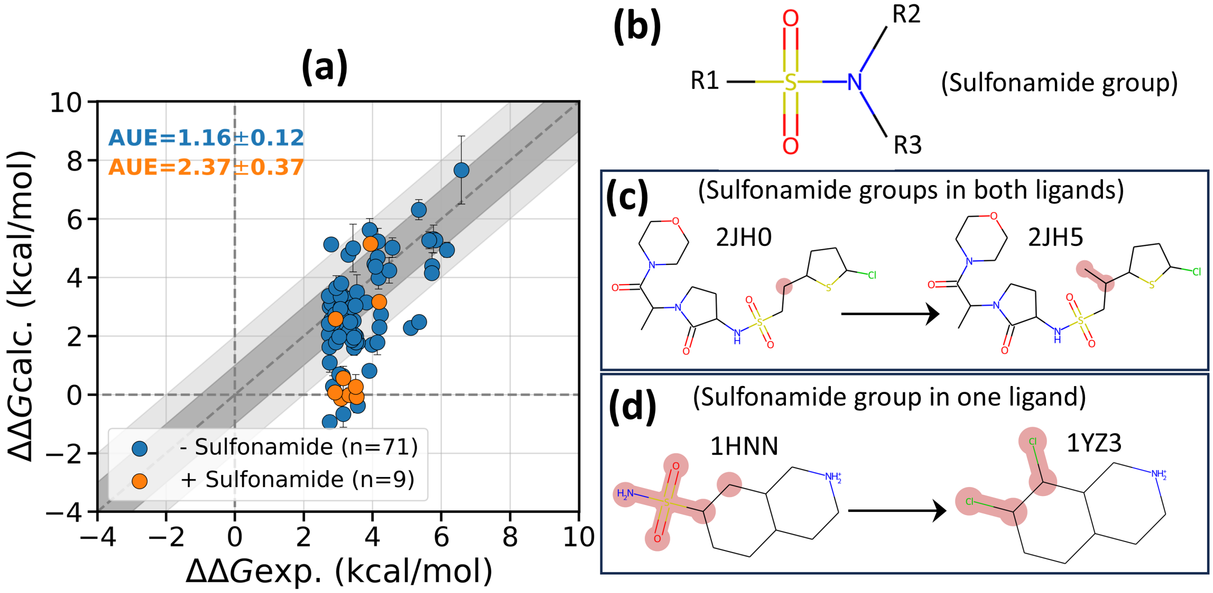

Figure S10: Impact of sulfonamide groups on RBFE calculation accuracy. (a) Comparison of prediction errors for ligand pairs with ("+ Sulfonamide") and without ("- Sulfonamide") sulfonamide groups. Data points are from the "Best set", with 'n' indicating sample size. (b) Molecular picture of a sulfonamide group. Examples of ligand transformations: (c1) both ligands contain sulfonamide groups, (c2) sulfonamide is present in only one ligand. Perturbation sites are highlighted in light red, with corresponding PDB IDs shown.

Further analysis of the "Best set" reveals several data points with errors $\geq$ 2 kcal/mol (Figure S4a). Notably, many of these high-error cases involve ligands containing a sulfonamide group (Figure S10b). To investigate this trend, we divided the "Best set" into two subsets: "+ Sulfonamide," where at least one ligand contains a sulfonamide group, and "- Sulfonamide," where neither ligand contains this group. The presence of a sulfonamide group leads to larger errors (Figure S10a), as evidenced by the higher AUE in the "+ Sulfonamide" subset (2.37 $\pm$ 0.37 kcal/mol) compared to the "- Sulfonamide" subset (1.16 $\pm$ 0.12 kcal/mol). Although the "+ Sulfonamide" subset is relatively small (n=9), it highlights the challenges in accurately capturing $\Delta\Delta G$ values for ligands containing sulfonamide groups. This observation aligns with previous studies[4–6] that have reported higher errors in free energy estimates for ligand transformations involving sulfonamide groups, attributing

these discrepancies to force field parameters. Our findings of increased errors likely share common origins with previous studies. These errors could potentially be mitigated through systematic refinement of force field parameters. These findings highlight the need for special consideration when dealing with sulfonamide-containing ligands in free energy calculations.

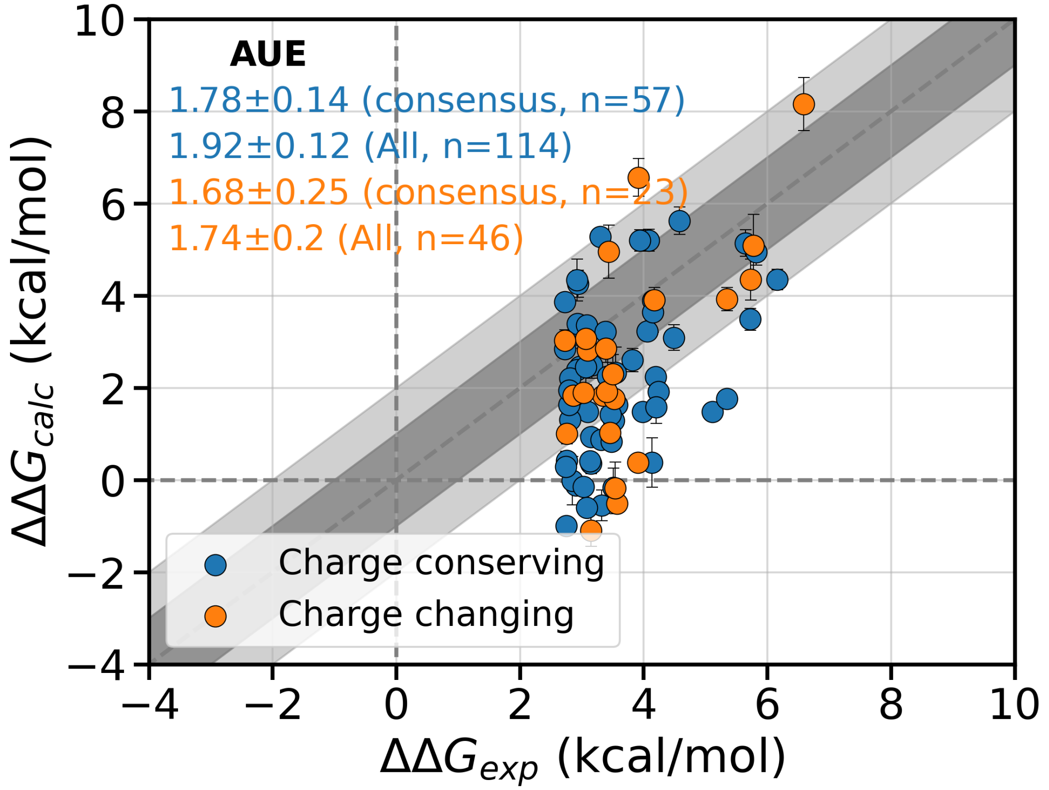

Figure S11: Comparison of charge-conserving mutations with charge-changing ones. Charge-changing mutations achieve comparable accuracy to charge-conserving ones, likely due to the longer transition time for the former (500 ps versus 200 ps). Only the consensus data points are plotted.

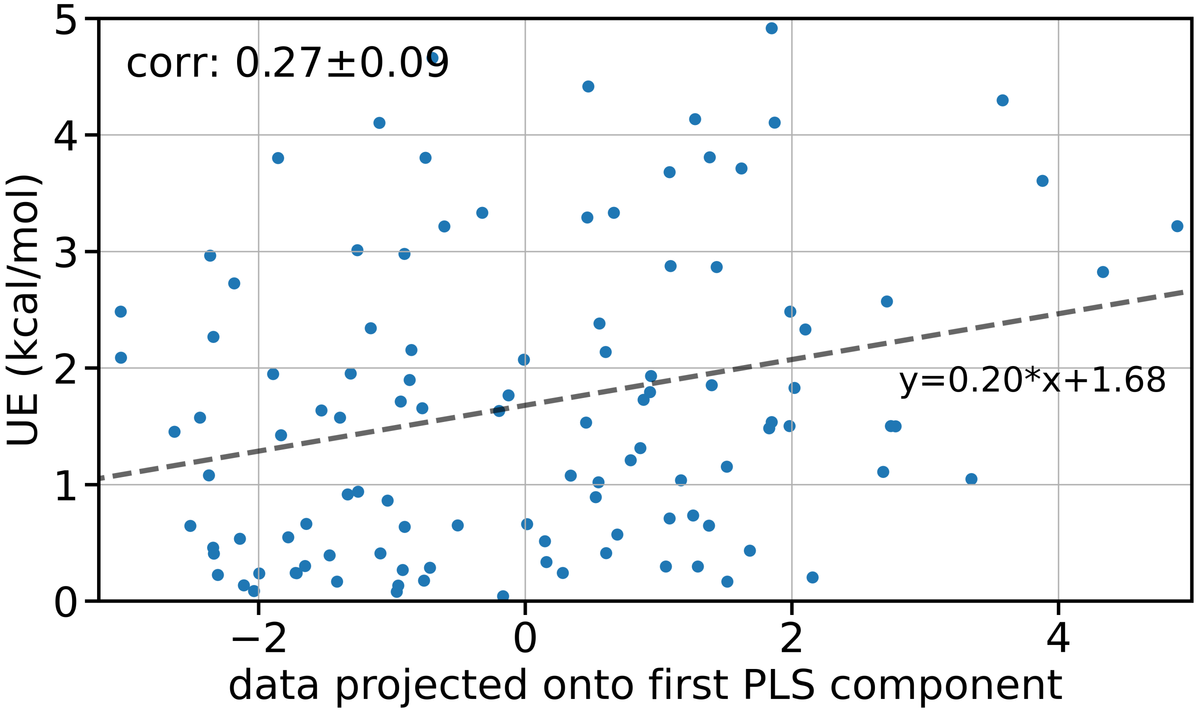

Figure S12: Partial Least Squares (PLS) regression analysis of crystal structure features and its relationship unsigned error (UE). The first PLS component, derived from five crystal structure features (resolution, packing score, number of water molecules, ligand B-factor, and protein B-factor), shows a Pearson correlation coefficient with UE comparable to individual features.

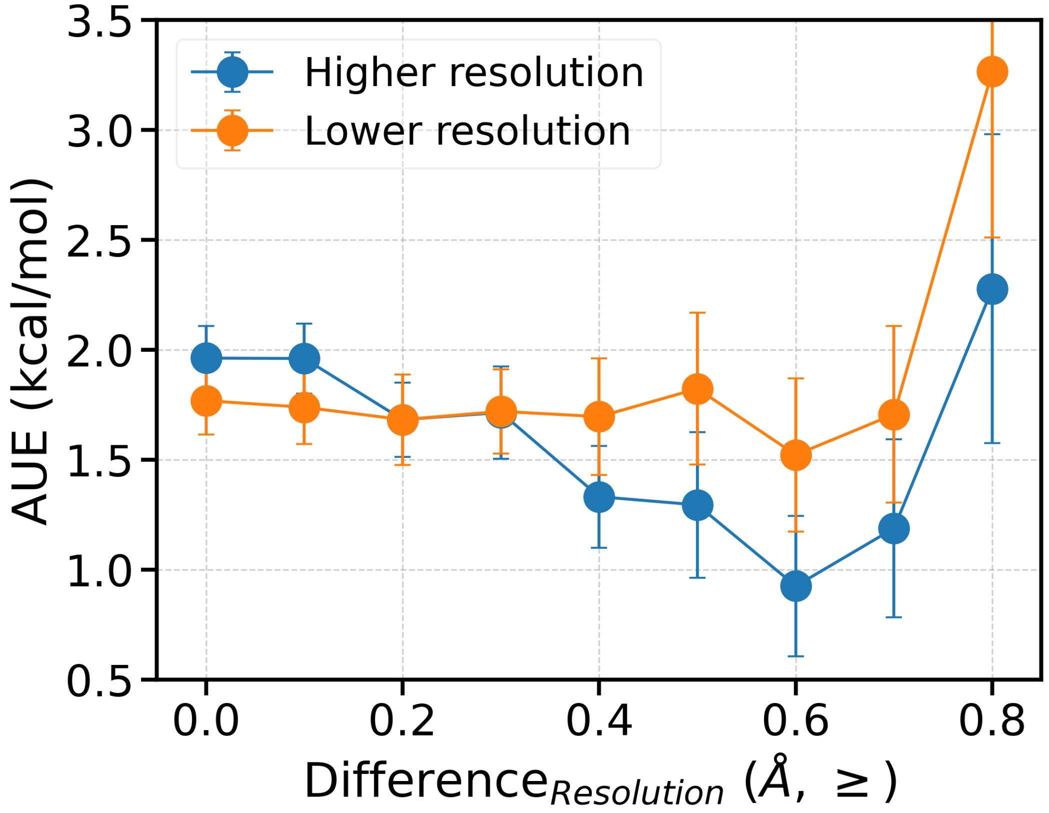

Figure S13: The two crystal structures for each ligand pair are categorized into 'Higher' (lower in Å) and 'Lower' (higher in Å) resolution sets. Plots show the change in AUE as a function of the minimum difference in resolution between the two sets.

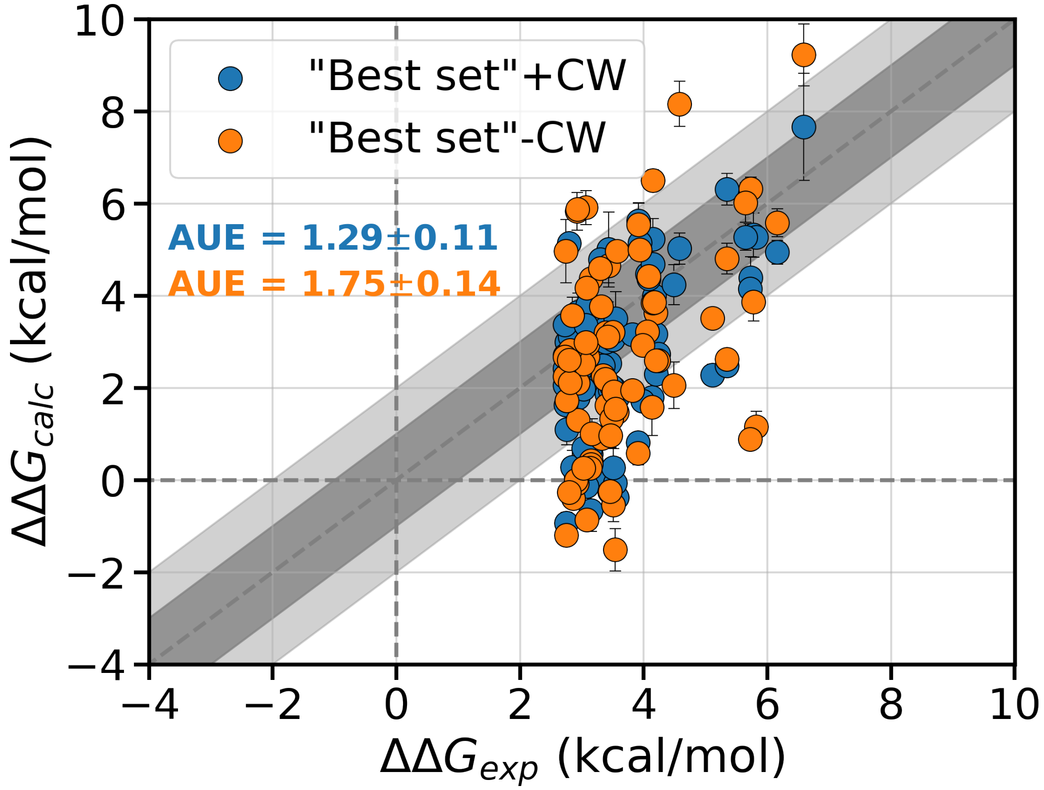

Figure S14: The ‘Best set’ with (+CW) and without (-CW) crystal water. The presence of crystal water molecules in the simulations has a significant impact on the accuracy of free energy estimates.

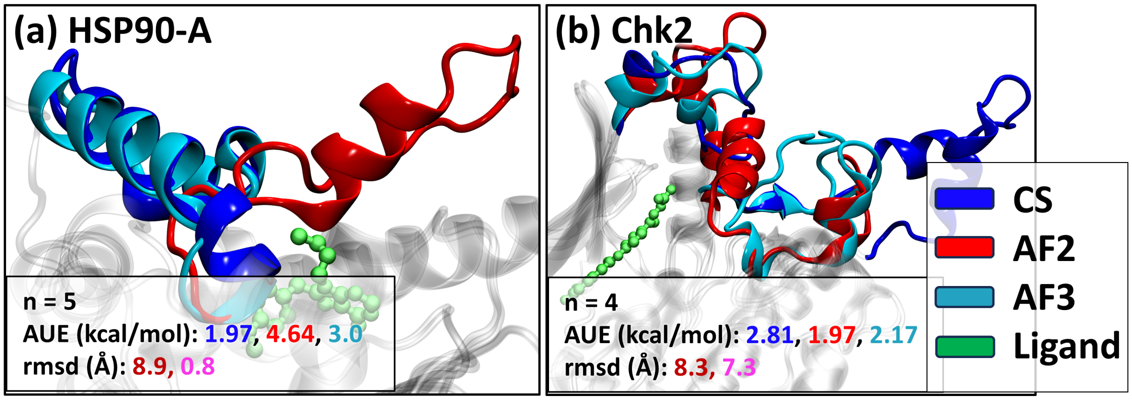

Figure S15: Similar to Figure 5 of the main text for two additional targets, HSP90-A and Chk2. The number of data points (n), AUE and rmsd of the highlighted regions with respect to CS for AF2 and AF3 are written. The uncertainty on AUE, derived as bootstrapped standard error, varies from 0.25 to 0.5 kcal/mol. The colour scheme is consistent across both the panels.

Table S1: Protein targets and their corresponding PDB ID pairs ('ID1_ID2') in the current activity cliff dataset.

Target	no. of ligand pairs	pdbid pairs
Thrombin	23	[2ZDA_2ZHQ, 2ZDA_3P17, 3RLW_2ZHQ, 2ZO3_3DHK, 2ZC9_3SI4, 2ZC9_3QTV, 2ZDA_3SI4, 2ZDA_3SV2, 2ZDA_2ZFF, 2ZDA_3QTV, 2ZDA_3SI3, 2ZHQ_3SI4, 2ZC9_3QTO, 2ZDA_3QTO, 2ZNK_2ZI2, 2ZC9_3P17, 2ZDA_3QWC, 2ZDA_3QX5, 2JH0_2JH5, 2ZC9_3SI3, 1O2G_1GJ4, 2ZC9_3SV2, 1TA2_3SHC]
Cyclin-dependent kinase 2	10	[1H1S_1H1R, 2UZB_2UZE, 1H1S_1H1Q, 2R3J_2R3I, 2UZN_2UZO, 2VTN_2VTL, 2R3M_2R3I, 2R3J_2C68, 2UZD_2UZE, 1H1S_1OGU]
Leukotriene A4 hydrolase	6	[3FH8_3FUM, 3FH8_3FUL, 3FH5_3FUM, 3FUI_3FUM, 3FH5_3FUL, 3U9W_3FUM]
Heat shock protein HSP 90-alpha	5	[2FWZ_1UY9, 2FWY_1UY7, 2FWY_1UY8, 2FWZ_1UYK, 2FWY_1UYC]
Beta-secretase 1	5	[2VJ6_2VIY, 3LHG_4FRI, 3RSV_3RTN, 4B1D_4FRI, 3LHG_4DJV]
Serine/threonine-protein kinase Chk2	4	[2YIT_2W7X, 2XK9_2W7X, 2YCQ_2W7X, 2YIR_2W7X]
Serine/threonine-protein kinase Aurora-A	3	[3UOH_4DEA, 3UOK_4DEA, 3UO6_4DEA]
Phenylethanolamine N-methyltransferase	3	[1YZ3_1HNN, 1YZ3_3HCC, 1YZ3_1N7I]
Renin	2	[3OOT_3OQK, 3GW5_3Q5H]
Coagulation factor X	2	[1MQ6_1MQ5, 2FZZ_3KQE]
Serine/threonine-protein kinase Chk1	2	[4FSZ_4FSW, 2YEX_2YER]
Urokinase-type plasminogen activator	2	[1OWD_1OWE, 1C5X_1C5Y]
Methionine aminopeptidase 2	2	[2EA2_2GA2, 1YW8_1YW7]
Glutamate carboxypeptidase II	2	[3D7H_3SJF, 3D7H_3IWW]

Target	no. of ligand pairs	pdbid pairs
Serine/threonine-protein kinase PIM1	1	[3UMW_3UMX]
Vascular endothelial growth factor receptor 2	1	[3CP9_3CPC]
Glutamate receptor ionotropic kainate 1	1	[3FVK_3FVN]
PI3-kinase p110-gamma subunit	1	[3L08_3S2A]
Hypoxanthine-guanine phosphoribosyltrans-ferase	1	[3GGJ_3GEP]
Matrix metalloproteinase 13	1	[1XUD_1XUR]
Protein-tyrosine phosphatase 1B	1	[2CM8_2BGE]
Serine/threonine-protein kinase NEK2	1	[2XNM_2XNN]
Adenosine kinase	1	[2I6A_1BX4]

References

(1) Gapsys, V.; Pérez-Benito, L.; Aldeghi, M.; Seeliger, D.; Van Vlijmen, H.; Tresadern, G.; De Groot, B. L. Large scale relative protein ligand binding affinities using non-equilibrium alchemy. *Chemical Science* **2020**, *11*, 1140–1152.

(2) Pérez-Benito, L.; Casajuana-Martin, N.; Jiménez-Rosés, M.; Van Vlijmen, H.; Tresadern, G. Predicting activity cliffs with free-energy perturbation. *Journal of Chemical Theory and Computation* **2019**, *15*, 1884–1895.

(3) Khalak, Y.; Tresadern, G.; Aldeghi, M.; Baumann, H. M.; Mobley, D. L.; de Groot, B. L.; Gapsys, V. Alchemical absolute protein–ligand binding free energies for drug design. *Chemical Science* **2021**, *12*, 13958–13971.

(4) Hahn, D. F.; Gapsys, V.; de Groot, B. L.; Mobley, D. L.; Tresadern, G. Current state of open source force fields in protein–ligand binding affinity predictions. *Journal of Chemical Information and Modeling* **2024**, *64*, 5063–5076.

(5) Aldeghi, M.; Heifetz, A.; Bodkin, M. J.; Knapp, S.; Biggin, P. C. Predictions of ligand selectivity from absolute binding free energy calculations. *Journal of the American Chemical Society* **2017**, *139*, 946–957.

(6) Christ, C. D.; Fox, T. Accuracy assessment and automation of free energy calculations for drug design. *Journal of Chemical Information and Modeling* **2014**, *54*, 108–120.
